# Lower rates of return to sport in patients with generalised joint hypermobility two years after ACL reconstruction: a prospective cohort study

**DOI:** 10.1186/s13102-023-00707-2

**Published:** 2023-08-12

**Authors:** Jakob Lindskog, Ramana Piussi, Rebecca Simonson, Johan Högberg, Kristian Samuelsson, Roland Thomeé, David Sundemo, Eric Hamrin Senorski

**Affiliations:** 1 Sportrehab Sports Medicine Clinic, Stampgatan 14, Gothenburg, SE-411 01 Sweden; 2 Unit of Physiotherapy, Department of Health and Rehabilitation, Institute of Neuroscience and Physiology, Sahlgrenska Academy, University of Gothenburg, Box 455, Gothenburg, SE-405 30 Sweden; 3 Sahlgrenska Sports Medicine Center, Sahlgrenska Academy, Gothenburg, Sweden; 4 Department of Orthopaedics, Institute of Clinical Sciences, Sahlgrenska Academy, University of Gothenburg, Gothenburg, Sweden; 5 Department of Orthopaedics, Sahlgrenska University Hospital, Mölndal, Sweden; 6 Närhälsan Lerum Primary Health Care Center, Lerum, Sweden

**Keywords:** Anterior cruciate ligament reconstruction, Return to sport, Generalised joint hypermobility, Physical evaluation

## Abstract

**Background:**

Generalised joint hypermobility (GJH) has been associated with an increased risk of suffering an anterior cruciate ligament (ACL) injury. Patients with GJH exhibit lower muscle strength and poorer scores for patient-reported outcomes after ACL reconstruction, compared with patients without GJH. The aim of this study was to examine differences in the percentages of patients who return to sport (RTS) or pre-injury level of activity (RTP), muscle function and patient-reported outcomes at the time of RTS or RTP, as well as the time of RTS or RTP in patients with GJH compared with patients without GJH in the first two years after ACL reconstruction.

**Methods:**

This prospective study used data from an ACL- and rehabilitation-specific register located in Gothenburg, Sweden. Patients aged between 16 and 50, who had a primary ACL injury treated with reconstruction, were included. Data up to two years after ACL reconstruction were used and consisted of achieving RTS and RTP, results from isokinetic muscle function tests for knee extension and flexion and patient-reported outcomes (Knee Self-Efficacy Scale, Knee injury and Osteoarthritis Outcome Score and ACL-Return to Sport after Injury scale) at the time of RTS, as well as the time of RTP. A Beighton Score of ≥ 5/9 was used to define GJH. A Tegner Activity Scale of ≥ 6 was used to define RTS, while a Tegner equal to or above pre-injury level was used to define RTP.

**Results:**

A total of 1,198 patients (54.7% women) with a mean age of 28.5 ± 8.6 years were included. A smaller proportion of patients with GJH achieved RTS compared with patients without GJH (49.2% vs. 57.3%, Odds ratio: 0.720, *p* = 0.041). Furthermore, patients with GJH were marginally less symmetrical on the knee extension strength test, expressed as a Limb Symmetry Index, at the time of RTP compared with patients without GJH (87.3 ± 13.5 vs. 91.7 ± 14.3, Cohen’s d = 0.142, *p* = 0.022). No further differences were found between groups regarding any muscle function tests or patient-reported outcomes.

**Conclusion:**

A smaller proportion of patients with GJH achieved RTS compared with patients without GJH. Patients with GJH displayed less symmetrical knee extension strength at the time of RTP compared with patients without GJH.

**Supplementary Information:**

The online version contains supplementary material available at 10.1186/s13102-023-00707-2.

## Introduction

Generalised joint hypermobility (GJH) is a hereditary condition that results in an exaggerated ability to move body joints beyond the normal range of motion [1]. The prevalence of GJH ranges from 2 to 57% and the condition is more prevalent in women and the younger population [2–4]. Common methods for identifying GJH are the Beighton Score [5], the Carter and Wilkinson criteria [6] and the five-part questionnaire on hypermobility [7]. Historically, no universal agreement for defining GJH has been reached, although the Beighton Score [5], defined by nine joint tests in which the thumbs, little fingers, elbows, knees and back are tested (Table 1), is the most frequently used in scientific literature [8]. For men and women at pubertal age up to the age of 50 years, five positive tests from the Beighton score, i.e. moving five joints beyond the normal range of motion, are defined as having GJH according to global scientific consensus [9].

The presence of GJH has been associated with an increased risk of sustaining a primary knee injury [10], including anterior cruciate ligament (ACL) injury [11]. Additionally, women are at greater risk for ACL injury compared to men [12, 13]. Upon suffering an ACL injury, the preferred treatment for patients who intend to return to a jumping, cutting or pivoting sport is surgical reconstruction of the ACL followed by structured rehabilitation [14]. Reconstructive surgery in Sweden is most often performed with a tendon autograft from the hamstrings (HT), patella (PT) or quadriceps (QT) [15]. Following ACL reconstruction, patients with GJH show increased knee laxity and achieve lower subjective knee scores, regardless of graft choice, compared with patients with normal mobility [16, 17]. However, according to previous research, patients with GJH, treated with PT, display less postoperative knee laxity and superior scores on patient-reported outcomes (PROs) in terms of knee function and symptoms compared with patients with GJH treated with HT [10, 18–20].

Rehabilitation after ACL reconstruction aims to recover muscle strength and function for a safe return to sport (RTS) [10], i.e. RTS with the lowest possible risk of sustaining a second knee injury (2KI) [14, 21]. However, only about 50% of patients RTS between one and two years after ACL reconstruction [16, 22]. Of these 50%, up to 30% go on to suffer a 2KI including a second ACL injury [17] or meniscal or cartilage injuries [23]. In order to minimise the risk of a 2KI, it has been suggested that various factors should be considered for a safe RTS [24]. These factors include, but are not limited to, muscle strength and function, PROs and time to RTS [25]. Firstly, ≥ 90% symmetrical quadriceps strength (injured leg compared with non-injured leg) has been reported as having a protective value against 2KI [26], but the results are not always confirmed [27]. Secondly, with regard to PROs, both a relatively negative psychological response (60.8 on the ACL-Return to Sport after Injury (ACL-RSI)) scale and a positive (81.2 on the ACL-RSI) psychological response have been associated with a risk of a second ACL injury [28, 29]. Finally, Beischer et al. [30] found that patients who RTS < 9 months after ACL reconstruction run a seven times higher risk of suffering a second ACL injury compared with patients who RTS > 9 months after ACL reconstruction, but the results are not always reproduced [31].

As inferior postoperative outcomes have been found in patients with GJH following ACL reconstruction when compared with patients without GJH [16, 17], the presence of GJH might also influence RTS after ACL reconstruction. There is an evident need for further knowledge of the impact of GJH on achieving RTS, muscle function tests and PROs at RTS, as well as time to RTS, in patients with GJH who RTS following ACL reconstruction.

Therefore, the aim of this study was primarily to analyse RTS and secondly return to pre-injury level of activity (RTP) in patients with GJH compared with patients without GJH, after ACL reconstruction.

## Method

This was a prospective study, conducted according to the REporting of studies Conducted using Observational Routinely-collected health Data (RECORD) statement [32]. Data for the present study were extracted on 22 November 2022 from a rehabilitation-specific register: Project ACL, which started in 2014 and is located in Gothenburg, Sweden. Project ACL contains data on patients with an ACL injury and has previously been described in detail [33, 34]. Patients registered in Project ACL are evaluated according to a predefined follow-up schedule at 10 weeks, four, eight, 12 and 18 months, two years and every five years after baseline, i.e., ACL injury or reconstruction. Patients are evaluated with both validated muscle function tests and PROs. Informed consent was obtained from patients at time for registration in Project ACL. After approval of the register holder, data was extracted by the first author. All methods were carried out in accordance with the declaration of Helsinki. Ethical approval was obtained from the Regional Ethical Review Board in Gothenburg, Sweden (registration numbers: 265–13, T023–17).

### Muscle function tests

The tests of muscle function included strength tests for the quadriceps and hamstring muscle groups, as well as hop performance tests. Muscle strength tests for knee extension and flexion were performed in an isokinetic dynamometer; Biodex System 4 (Biodex Medical Systems, Shirley, New York, USA) [35] at an angular velocity of 90°/second. The Biodex dynamometer has good instrumental validity [36] (intraclass correlation coefficient (ICC) = 0.99-1.00) and test-retest reliability [37] (ICC = 0.95) when measuring strength in knee extension and knee flexion reflecting quadriceps and hamstring strength. The following test procedure protocol has previously been described in detail [38]. Prior to the tests of muscle function, patients performed a standardised warm-up of 10 min on a stationary bike. Following the standardised warm-up, the Beighton Score [5] was assessed. The Beighton Score was added to Project ACL in 2019 and is registered in the database as a total score. Alongside the Beighton Score, the presence of knee hyperextension (yes/no) is also registered in the database. Knee extension strength was measured from 90−0° of knee flexion and knee flexion strength was measured from 0–90° of knee flexion. Patients were tested in a seated position with straps around the torso and the leg that was being tested. The injured leg was tested first. Prior to a maximum muscle strength test, patients familiarised themselves with the Biodex, starting with 10 repetitions at 50% of maximum effort (ME), 10 repetitions at 75% of ME and one repetition at 90% of ME. The maximum test was then performed through three separate one-ME repetition trials with a 40-second rest in between. The highest measured peak torque (Nm) was registered in Project ACL's database and used for the analysis of knee extension and flexion respectively.

Following the strength tests, hop tests were performed; they consisted of a vertical hop, hop for distance and the 30-second side-hop test. All the hop tests are valid and reliable for measuring hop performance in patients with an ACL injury [39]. Hop tests were first performed on the injured leg and patients were instructed to hold their hands behind their back throughout the tests. For the vertical hop, flight time was measured with the infrared optical contact grids 2 m apart from Muscle lab, Ergotest Technology, Oslo, Norway, converted into centimetres (cm). In the hop for distance, the distance in cm from the toe at take-off to the heel at landing was recorded with a parallel measuring tape integrated in the floor. For the vertical hop and the hop for distance, two to three warm-up hops were allowed before maximum performance. Three maximum hops were performed after warm-up and the best recorded result in cm was registered in Project ACL’s database and used for analysis. During the 30-second side-hop test, patients performed as many hops as possible for 30 s over two lines 40 cm apart. The total number of hops was counted, with a one-hop deduction for every hop not completely over one of the lines, and was then registered in Project ACL’s database and used for analysis. Ten warm-up hops were allowed before the maximum effort test.

The results of muscle function tests were reported and analysed with the LSI, which is calculated as followed: $\left(\frac{Result\ for\ injured\ leg}{Result\ for\ non-injured\ leg}\right)*100$, presented as a percentage.

### Patient-reported outcomes

The PROs used in this study comprised the Tegner Activity Scale (Tegner), the Knee Self-Efficacy Scale (K-SES), the Knee injury and Osteoarthritis Outcome Score (KOOS) and, from the eight-month Project ACL follow-up onwards, the ACL-RSI scale. All the PROs used in this study are self-reported questionnaires.

Alongside the standardised PROs, as of March 2018, Project ACL implemented an additional question with regard to whether the patients had returned to their pre-injury activity and, if the answer was yes, how long ago, measured in months. The answer to this specific question was used to calculate the time to return to sport measured in months.

The Tegner aims to classify the patient’s level of knee-demanding activity from 0 (lowest knee-demanding activity) to 10 (highest knee-demanding activity) [40]. The Tegner used in this study was a modified version [33], in which the value “0”, representing “sick leave or disability pension because of knee problems”, was removed. In this study, RTS was defined as a return to level ≥ 6 on the Tegner [40], equalling activities like badminton, tennis, skiing or floorball, as well as active participation in sports such as baseball, snowboarding and hurdling. At Tegner level 6, no work activities are listed and the patients answering ≥ 6 are therefore expected to perform sports. Additionally, RTP was defined as reporting Tegner equal to or above pre-injury. Test-retest reliability: ICC = 0.8 for patients with an ACL injury and ACL reconstruction [41].

The K-SES aims to evaluate knee-related self-efficacy in patients with an ACL injury and consists of 18 items. The scale comprises two subscales, present and future [42], and the present was used in this study. Each item is scored on a 11-point Likert scale from 0 (poor self-efficacy) to 10 (strong self-efficacy). Only the K-SES present subscale was analysed in this study. The K-SES has sufficient construct validity tested as structural validity, hypothesis testing and cross-cultural adaptation [42]. Test-retest reliability: ICC = 0.92 and internal consistency: Cronbach’s α = 0.81–0.96 [42].

The KOOS aims to evaluate subjective knee function divided into five subscales: pain, symptoms, activity of daily living, function in sports and recreation and quality of life (QoL) [43]. Patients answer the questions with respect to the previous week. Each question is scored on a five-point Likert scale from 0 (maximum negative response) to 4 (maximum positive response). The answers are recalculated to produce a normalised score ranging from 0 (severe knee-related symptoms/QoL) to 100 (no knee-related symptoms/QoL) [43]. The KOOS has test-retest reliability: ICC = 0.78-0.97 [43] and internal consistency: Cronbach’s α = 0.70-0.95 [44] and only the function on the sports and recreation subscale exhibits acceptable construct validity tested as > 75% confirmation of a predefined hypothesis [45]. The KOOS has not been validated for evaluation after an ACL injury, but it is often used for evaluation after ACL reconstruction [46] and was therefore included in this study. As the items on the KOOS subscale activity of daily living are not aimed at sports participation, the subscale was not analysed in this present study.

The ACL-RSI aims to assess emotions, confidence and risk appraisal in relation to RTS [47]. The 12-item version was used in this study [48]. The items are scored from 1 (lowest emotion, confidence and risk appraisal in relation to RTS) to 10 (highest emotion, confidence and risk appraisal in relation to RTS) [47, 49]. The score is presented as a normalised score from 10 (lowest emotion, confidence and risk appraisal in relation to RTS) to 100 (highest emotion, confidence and risk appraisal in relation to RTS). The ACL-RSI has internal consistency: Cronbach’s α = 0.95, relevant face validity tested in a discussion with experts and patients, construct validity tested as hypothesis testing [47] and the ability to predict RTS is fair to good [47, 50].

### Patients

Patients registered in Project ACL who were between 16 and 50 years of age at the time of ACL reconstruction, who had suffered an ACL injury treated with reconstruction and had follow-up data on one of Project ACL’s follow-ups from four months to two years were eligible for inclusion. Patients who did not have data registered for GJH, Tegner, or had suffered more than one ACL injury were excluded from this study.

Data from all the follow-ups in Project ACL between four months after ACL reconstruction and the two-year follow-up were extracted for analysis, containing demographics such as sex, age, height, weight, days between injury and surgery, time to RTS, details of surgical treatment (graft choice), presence of knee hyperextension (HE), GJH, tests of muscle function and patient-reported outcomes.

### Definition of study groups

In the present study, patients were divided into two groups: patients with a Beighton score [5] of ≥ 5 comprised the GJH group, while patients with a Beighton score [5] of ≤ 4 comprised the non-GJH group (Table 1).

**Table 1:** Beighton score

Joint test	Left	Right
Passive dorsiflexion of the metacarpal joint of the fifth finger beyond 90°	Yes/No	Yes/No
Passive apposition of the thumbs to the flexor aspects of the forearms	Yes/No	Yes/No
Passive hyperextension of the elbows beyond 10°	Yes/No	Yes/No
Passive hyperextension of the knees beyond 10°	Yes/No	Yes/No
Active forward flexion of the trunk, with the knees straight, so that the palms of the hands rest easily on the floor	Yes/No	

### Outcomes

The primary outcome of this study was the proportion of patients who RTS (defined as Tegner ≥ 6), the comparison of the muscle function tests and the K-SES [42], KOOS [43] and ACL-RSI [47, 48] at the time of RTS and the time to RTS in each of the study groups: the GJH group and the non-GJH group.

The secondary outcome was the proportion of patients who RTP (defined as Tegner equal to or above pre-injury), the comparison of the muscle function tests and the K-SES [42], KOOS [43] and ACL-RSI [47, 48] at the time of RTP and the time to RTP in each of the study groups: the GJH group and the non-GJH group.

### Statistical analyses

Statistical analyses were performed with the Statistical Product and Service Solutions (IBM Corp. Released 2017. IBM SPSS Statistics for Windows, Version 25.0. Armonk, NY: IBM Corp.). The results were presented stratified by group. The difference in the proportion of patients who achieved RTS and RTP was analysed using Fisher’s exact test across all follow-ups from four months to two years presented as count with percent and an odds ratio (OR) with 95% confidence interval (CI). Muscle function tests, K-SES, KOOS and ACL-RSI were analysed using the independent t-test, while Tegner was analysed using the Mann-Whitney U test at the time of RTS and RTP respectively. The group difference in terms of time to RTS and time to RTP, measured in months, was analysed using the independent t-test. Demographic data were analysed using an independent t-test for continuous variables and Fisher’s exact test for categorical variables for each of the primary and secondary outcomes. To evaluate the significance of differences, Cohen’s d was calculated and the following reference values were used: 0.20 = small, 0.50 = medium and 0.80 = large [51]. The significance level was set at 95%. In the event of an analysis showing a statistically significant result, CIs and Cohen’s d were also reported.

## Results

**Fig. 1:**
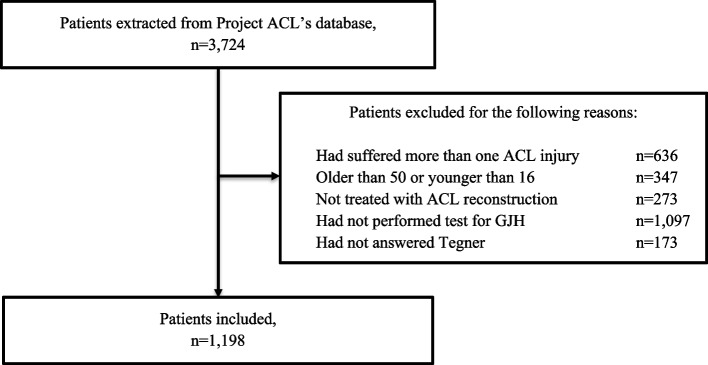
Flowchart of included and excluded patients, GJH = Generalised Joint Hypermobility, ACL = Anterior Cruciate Ligament, *n* = number of patients, Tegner = Tegner Activity Scale

A total of 3,724 patients were extracted from Project ACL, of which 1,198 patients were included in this study (Fig. 1). Included patients’ ACL reconstructions were performed between 24 April 2013 and 12 July 2022. Patient demographics on included patients are described in Table 2.

**Table 2:** Demographic data for all the included patients

Patient demographics	Total, *n* = 1,198	GJH, *n* = 183	Non-GJH, *n* = 1,015	*p*-value
Sex, women, n (%)	665 (54.7)	143 (78.1)	512 (50.4)	< 0.001^*^
Age, years, mean ± SD	28.5 ± 8.6	26.6 ± 8.4	28.9 ± 8.6	< 0.001^*^
Height, cm, mean ± SD	174.1 ± 8.8	171.6 ± 8.7	174.5 ± 8.8	< 0.001^*^
Weight, kg, mean ± SD	72.5 ± 12.2	69.3 ± 11.9	73.1 ± 12.2	< 0.001^*^
Time between injury and surgery, days, mean ± SD	303 ± 641	315 ± 817	301 ± 605	0.789
Knee HE, n (%) missing, [n]	306 (30.9) [209]	139 (80.8) [11]	167 (20.4) [198]	< 0.001^*^
Graft choice, n (%)				
HT	911 (77.7)	122 (69.3)	789 (79.2)	0.019^*^
PT	235 (20.1)	51 (29.0)	184 (18.5)	
Allo	6 (0.5)	0	6 (0.6)	
QT	4 (0.3)	1 (0.6)	3 (0.3)	
Other	16 (1.4)	2 (1.1)	14 (1.4)	
Missing, [n]	[26]	[7]	[19]	
Tegner pre-injury, median (min-max)	8 (1–10)	8 (1–10)	8 (1–10)	0.459

*n *number of patients, *GJH *Generalised Joint Hypermobility, *kg* kilogram, *cm *centimetres, *HE *Hyperextension, *HT *Hamstring tendon, *PT *Patellar tendon, *QT *Quadriceps tendon, *Tegner *Tegner Activity Scale, *SD *Standard deviation, ^*^= statistically significant difference (*p*<0.05)

### Primary outcomes

#### Proportions of patients who RTS, muscle function tests and patient-reported outcomes at RTS

Of the 1,198 included patients (GJH *n* = 183, non-GJH *n* = 1,015), there was a significantly smaller proportion of patients who achieved RTS within two years after ACL reconstruction in the GJH group compared with the non-GJH group (90/183, 49.2% vs. 582/1,015, 57.3%, OR: 0.720 [95% CI: 0.525, 0.987] respectively, *p* = 0.041) (Table 3). An ad-hoc analysis was performed on RTS stratified by patient sex and graft choice, respectively, and showed no statistical difference depending on these variables.

Of the 672 patients (GJH *n* = 90, non-GJH *n* = 582) who achieved RTS, a varying number of patients performed muscle function tests and answered PROs (Table S1). There was no statistically significant difference between the GJH and non-GJH groups regarding the results of muscle function tests and PROs at the time of RTS (Table S2).

In patients who RTS, the GJH group, compared with the non-GJH group, consisted of a larger proportion of women (67/90, 74.4% vs. 286/582, 49.1% respectively, *p* < 0.001), were younger (24.2 ± 7.2 vs. 27.3 ± 8.2 years respectively, *p* < 0.001), had a larger proportion of knee HE (71/87, 81.6% vs. 92/493, 18.7% respectively, *p* < 0.001) and a larger proportion of PT grafts used for ACL reconstruction (30/90, 33.3% vs. 118/578, 20.4% respectively, *p* = 0.038) (Table 3). Patients included in the primary analysis did not differ in terms of patient demographics compared with the total cohort.

### Time to RTS

Of the 672 patients who RTS, 114 patients (GJH *n* = 13, non-GJH *n* = 101) reported the time to RTS (Table 3). No significant difference regarding the time to RTS was found. The GJH group reported 10.8 ± 4.1 months and the non-GJH group 12.1 ± 3.9 months (*p* = 0.258).

In patients who reported the time to RTS, the GJH group, compared with the non-GJH group, were younger (22.5 ± 3.6 vs. 28.3 ± 8.4 years respectively, *p* = 0.016) and had a larger proportion of knee HE (10/11, 90.9% vs. 16/81, 19.8% respectively, *p* < 0.001) (Table 3). No further demographic difference was found.

**Table 3:** Proportions of patients who RTS, time to RTS and demographic differences in patients

Patient demographics	Patients who RTS			Patients who reported time to RTS		
	**GJH**, *n* = 90	**Non-GJH**, *n* = 582	***p***-value	**GJH**, *n* = 13	**Non-GJH**, *n* = 101	***p***-value
**Time to RTS**, months, mean ± SD	-	**-**	**-**	10.8 ± 4.1	12.1 ± 3.9	0.258
**Proportion of patients who RTS**, n/total (%)	90/183 (49.2)	582/1015 (57.2)	0.043*	**-**	**-**	**-**
Sex, women, n (%)	67 (74.4)	286 (49.1)	< 0.001*	10 (76.9)	55 (54.5)	0.147
Age, years, mean ± SD	24.2 ± 7.2	27.3 ± 8.2	< 0.001*	22.5 ± 3.6	28.3 ± 8.4	0.016^*^
Height, cm, mean ± SD	173.0 ± 9.0	174.8 ± 8.7	0.088	172.8 ± 8.6	173.6 ± 8.8	0.741
Weight, kg, mean ± SD	70.1 ± 12.5	72.1 ± 11.6	0.135	69.7 ± 11.7	70.3 ± 10.0	0.841
Time between injury and surgery, days, mean ± SD	191 ± 185	226 ± 434	0.224	139 ± 168	310 ± 644	0.344
Knee HE, n (%) missing, [n]	71 (81.6) [3]	92 (18.7) [89]	< 0.001*	10 (90.9) [2]	16 (19.8) [20]	< 0.001^*^
**Graft choice**, n (%)						
HT	59 (65.6)	452 (78.2)	0.038*	8 (61.5)	81 (81.0)	0.145
PT	30 (33.3)	118 (20.4)		5 (38.5)	19 (19.0)	
Allo	0	1 (0.2)		0	0	
QT	1 (1.1)	2 (0.3)		0	0	
Other	0	5 (0.9)		0	0	
Missing, [n]	[0]	[4]		[0]	[1]	
Tegner pre-injury, median (min-max)	9 (3–10)	8 (1–10)	0.569	9 (5–10)	8 (3–10)	0.115
Tegner at RTS, median (min-max)	7 (6–10)	7 (6–10)	0.821	8 (6–10)	7 (6–10)	0.075
**Tegner at RTS**, n (%)						
Level 1–5	0	0	0.883	0	0	0.342
Level 6	25 (27.8)	154 (26.5)		1 (7.7)	29 (28.7)	
Level 7	28 (31.1)	196 (33.7)		4 (30.8)	32 (31.7)	
Level 8	11 (12.2)	87 (14.9)		2 (15.4)	13 (12.9)	
Level 9	18 (20.0)	101 (17.4)		5 (38.5)	21 (20.8)	
Level 10	8 (8.9)	44 (7.6)		1 (7.7)	6 (5.9)	

*RTS *Return to Sport, *n *number of patients, *GJH *Generalised Joint Hypermobility, *kg *kilogram, *cm* centimetres, *HE *Hyperextension, *HT *Hamstring tendon, *PT *Patellar tendon, *QT *Quadriceps tendon, *Tegner *Tegner Activity Scale, *SD *Standard deviation, ^*^= statistically significant difference﻿ (*p*<0.05)

### Secondary outcomes

#### Proportions of patients who RTP, muscle function tests and patient-reported outcomes at RTP

Of the 1,198 included patients (GJH *n* = 183, non-GJH *n* = 1,015), there was no significant difference in the proportion of patients who RTP within two years after ACL reconstruction. The proportion in the GJH group was 80/183 patients (43.7%) compared with 446/1,015 patients (43.9%) in the non-GJH group (*p* = 1.000) (Table 4).

Of the 526 patients (GJH *n* = 80, non-GJH *n* = 446) who achieved RTP, a varying number of patients performed muscle function tests and answered PROs (Table S1). The non-GJH group had a higher quadriceps strength symmetry compared with the GJH group (91.7 ± 14.3 LSI, [95% CI = 90.2–93.1] vs. 87.3 ± 13.5 LSI, [95% CI = 83.9–90.3] respectively, *p* = 0.022, Cohen’s d = 0.142). There were no further statistically significant differences between groups in terms of the results of muscle function tests and PROs at the time of RTP (Table S2).

In patients who RTP, patients in the GJH group, compared with the non-GJH group, consisted of a larger proportion of women (58/80, 72.5% vs. 234/446, 52.5% respectively, *p* < 0.001), were younger (26.0 ± 8.6 vs. 28.6 ± 8.8 years respectively, *p* = 0.016), had a larger proportion of knee HE (60/78, 76.9% vs. 75/375, 20.0% respectively, *p* < 0.001) and a larger proportion of PT autografts used for ACL reconstruction (28/78, 35.9% vs. 83/443, 18.7% respectively, *p* = 0.004) (Table 4). No further demographic differences were found. Patients included in the secondary analysis did not differ in terms of patient demographics compared with the total cohort.

#### Time to RTP

Of the 526 patients who RTP, 110 patients (GJH *n* = 17, non-GJH *n* = 93) reported the time to RTP (Table 4). No significant difference was found between groups in terms of the time to RTP. The GJH group reported 11.7 ± 5.4 months and the non-GJH group 12.1 ± 4.0 months (*p* = 0.691).

In patients who reported the time to RTP, the GJH group, compared with the non-GJH group, had a larger proportion of knee HE (12/16, 75.0% vs. 14/75, 18.7% respectively, *p* < 0.001) (Table 4). No further demographic difference was found.

**Table 4:** Proportions of patients who RTP, time to RTP and demographic differences in patients

Patient demographics	Patients who RTP			Patients who reported time to RTP		
	**GJH**, *n* = 80	**Non-GJH**, *n* = 446	***p***-value	**GJH**, *n* = 17	**Non-GJH**, *n* = 93	***p***-value
**Time to RTP**, months, mean ± SD	-	**-**	**-**	11.7 ± 5.4	12.1 ± 4.0	0.691
**Proportion of patients who RTP**, n (%)	80/183 (43.7)	446/1015 (43.9)	1.000	**-**	**-**	**-**
Sex, women, n (%)	58 (72.5)	234 (52.5)	< 0.001*	12 (70.6)	51 (54.8)	0.291
Age, years, mean ± SD	26.0 ± 8.6	28.6 ± 8.8	0.016*	25.5 ± 7.8	28.6 ± 9.1	0.201
Height, cm, mean ± SD	173.6 ± 9.5	174.4 ± 8.5	0.442	175.0 ± 8.3	174.1 ± 8.7	0.692
Weight, kg, mean ± SD	72.7 ± 13.3	72.2 ± 11.5	0.720	75.9 ± 13.4	71.3 ± 11.6	0.140
Time between injury and surgery, days, mean ± SD	243 ± 472	266 ± 587	0.733	217 ± 287	254 ± 590	0.801
Knee HE, n (%) missing, [n]	60 (76.9) [2]	75 (20.0) [71]	< 0.001*	12 (75.0) [1]	14 (18.7) [18]	< 0.001^*^
**Graft choice**, n (%)						
HT	49 (62.8)	354 (79.9)	0.004*	10 (62.5)	77 (82.8)	0.088
PT	28 (35.9)	83 (18.7)		6 (37.5)	16 (17.2)	
Allo	0	1 (0.2)		0	0	
QT	1 (1.3)	1 (0.2)		0	0	
Other	0	4 (0.9)		0	0	
Missing, [n]	2	3		1	0	
Tegner pre-injury, median (min-max)	8 (1–10)	8 (1–10)	0.642	8 (3–10)	8 (1–10)	0.540
Tegner at RTP, median (min-max)	8 (2–10)	8 (1–10)	0.540	8 (3–10)	8 (2–10)	0.774
**Tegner at RTP**, n (%)						
Level 1	0	2 (0.4)	0.085	0	0	0.436
Level 2	3 (3.8)	8 (1.8)		0	3 (3.2)	
Level 3	9 (11.3)	16 (3.6)		2 (11.8)	1 (1.1)	
Level 4	4 (5.0)	35 (7.8)		0	4 (4.3)	
Level 5	4 (5.0)	26 (5.8)		1 (5.9)	7 (7.5)	
Level 6	6 (7.5)	22 (4.9)		1 (5.9)	10 (10.8)	
Level 7	6 (7.5)	69 (15.5)		1 (5.9)	13 (14.0)	
Level 8	15 (18.8)	94 (21.1)		4 (23.5)	18 (19.4)	
Level 9	23 (28.7)	107 (24.0)		7 (41.2)	25 (26.9)	
Level 10	10 (12.5)	67 (15.0)		1 (5.9)	12 (12.9)	

*RTP *Return to pre-injury level of activity, *n *number of patients, *GJH *Generalised Joint Hypermobility, *kg *kilogram, *cm *centimetres, *HE *Hyperextension, *HT *Hamstring tendon, *PT *Patellar tendon, *QT *Quadriceps tendon, *Tegner *Tegner Activity Scale, *SD *Standard deviation, ^* ^= statistically significant difference (*p*<0.05)

## Discussion

This study examined differences between frequencies of RTS and RTP, test of muscle function and PROs at time of RTS and RTP and time to RTS and RTP in patients with and without GJH up to two years following ACL reconstruction.

The main finding in this prospective observational register study was that a greater (16.5%) proportion of patients who, within two years after ACL reconstruction, returned to sport in the non-GJH group compared with the GJH group. The secondary findings were a more symmetrical quadriceps strength at the time of RTP in the non-GJH group compared with the GJH group and no difference in the mean time to RTS (10.8 ± 4.1 and 12.1 ± 3.9 months) or RTP (11.7 ± 5.4 and 12.1 ± 4.0 months) between groups.

### Main findings

In contrast to previously conducted meta-analyses which have reported a proportion of patients who RTS ranging from 73.2 to 83% [52–55] at six to 42 months after ACL reconstruction, the overall rate of RTS in the present study was lower and, in particular, in the GJH group (47.9%). This discrepancy in RTS rates can be influenced by the longer follow-up period [52–54] and many studies including highly active participants [52–55] compared with this study. Studies investigating highly active patients or patients performing competitive sports could yield a higher proportion of RTS, as previously reported by Mohtadi et al., [56] where up to 97% of National Hockey League players RTS. Project ACL aims to recruit all the patients who sustain an ACL injury, which means that patients at different levels on the physical activity scale, not solely at very high level, are present in the current study. In spite of this, the reason for the lower rate of RTS in the GJH group is not known. Other factors such as increased knee laxity [16, 17], or patients with GJH being more susceptible to knee instability may impact the ability to RTS for patients with GJH. Moreover, patients with GJH have been reported to experience more symptoms and poorer subjective knee function compared with patients without GJH [10, 18, 19], but this was not confirmed in this study. Accordingly, the PROs used in this study may not be sensitive enough to discriminate differences in instability between patients with and without GJH, despite the smaller proportion of RTS in the GJH group.

Taken together, this study showed a small difference in quadriceps LSI at the time of RTP in favour of the non-GJH group and no difference in the time to RTS or RTP, muscle function (except the quadriceps LSI difference at the time of RTP) or PROs at the time of RTS or RTP. The absence of differences between groups raises questions about what the potential consequences are for patients with GJH who RTS or RTP with the same clinical outcomes (knee strength symmetry and self-reported function) as patients without GJH. Patients with GJH run a higher risk of primary knee injury [10, 11] and 2KI [20, 57, 58]. In parallel, current RTS test batteries have shown inconsistency in identifying patients who run a higher risk of 2KI [59, 60]. This implies that there might be an even greater need for better reference values or recommendations for patients with GJH potentially to help reduce the risk of 2KI. Alternatively, other clinical tests that are more sensitive in mapping a higher 2KI risk for patients with GJH are needed. Similar arguments can be made about the time to RTS or RTP, since patients with GJH might benefit from a longer rehabilitation period to prepare adequately to RTS. Our recommendations for clinicians who rehabilitate patients with GJH after an ACL reconstruction would be to adopt open, clear communication relating to the possible risks of 2KI in relation to RTS or RTP.

### Proportions of patients who RTP, muscle function tests and patient-reported outcomes at RTS/RTP

In this study, there was no difference between groups in the proportion of patients who RTP. Similar results were reported by Ardern et al. [22] and DeFazio et al., [52] although these studies did not analyse GJH exclusively. As the distribution of Tegner levels did not differ between the groups in our study, this suggests that RTP is similar between patients with and without GJH. Consequently, GJH might not impact the ability to RTP, although clinicians should consider the greater risk of a 2KI [20, 57, 58].

There was no difference in quadriceps strength LSI between groups at the time of RTS, but quadriceps strength LSI was lower (4.8%) for the GJH group at the time of RTP compared with the non-GJH group. In the GJH group, in the RTP analysis, 35.9% of patients had the PT autograft as a choice for the ACL reconstruction, compared with 18.7% in the non-GJH group. Performing ACL reconstruction with PT autografts is recommended in patients with GJH [17, 61]. Harvesting the PT reduces quadriceps muscle strength for the first two years after ACL reconstruction [62, 63] and might partly explain the difference in LSI found in this study. In addition, Ewertowska et al. [64] found a lower peak quadriceps strength in patients with GJH, which was seen in patients with GJH at the time of RTP in this study. However, the difference in LSI between the GJH and non-GJH groups is fairly small and probably not clinically relevant and so, taking account of the Cohen’s d being only 0.142, caution is warranted when interpreting this result.

### Time to RTS/RTP

In this study, no difference in the time to RTS or RTP was found between the GJH group and the non-GJH group. This can be partly explained by the fact that the same patient can be included in both RTS and RTP analyses, i.e. reporting a Tegner of ≥ 6 and a Tegner equal to or above the pre-injury level. Great variability in the time to RTS is reported in the literature. Grindem et al. [26] reported a median RTS time of eight months (3–23 months), while Ardern et al. [22] reported that RTS can take up to two years, which agrees to some degree with the results in this study. There is no specific recommendation for the time to RTS for patients with GJH. Whether the time to RTS is a risk factor for a 2KI is still the subject of debate [14, 26, 65]. However, it could be argued that the increased laxity due to the GJH exposes patients with hypermobility to an increased risk of 2KI [57, 58]. However, this needs to be confirmed by future studies.

### Limitations

One limitation in this study is the differences in the distribution of patient sex between the groups, as women generally have GJH to a greater extent [2, 3]. The same pattern was observed in the current study, since 78.1% of patients with GJH were women. Furthermore, 54.7% of the included patients were women and the proportion of patients with GJH might therefore be lower than in the general population of patients who suffer an ACL injury. Consequently, the external validity of our results might be impacted. The difference in RTS rates in this study might be influenced by patient sex and not merely by the presence of GJH. An ad-hoc analysis on the rated of RTS was performed stratified by patient sex and graft choice, respectively. There analyses showed no statistical difference, hence, motivated our choice to examine differences between patients with and without GJH, independent of sex and graft. Additionally, the ages of studied group of patients differed with a mean difference of 2.3 years and was not considered clinically relevant to influence the result. The proportion of patients with GJH in Project ACL is small in relation to all the patients in the register, since the GJH outcome was added as late as 2019. This study only analysed the differences between patients with GJH and without GJH, regardless of patient sex. Furthermore, the aspect of graft choice could possibly have influenced the results in this study, since the proportions of PT and HT autografts differed between groups. However, the only difference seen in outcome was quadriceps strength LSI at the time of RTP. Moreover, an a priori sample size calculation based on the time to RTS was made. The calculation showed that 16 patients were needed, which was not fully achieved in the time to RTS in the GJH group (*n*= 13) and, as a result, this study is unable to discard the possibility of a difference in the time to RTS between groups. Sample size requirements regarding all the other outcomes were met. Furthermore, the time to RTS or RTP is susceptible to recall bias, since patients reported this retrospectively. Moreover, despite the fact that patients included in the analysis covered all the Tegner levels [1–10], most patients performed knee-strenuous sports (Tegner ≥ 6), indicating that the results might not be generalisable to the population not participating in knee-strenuous sports. In addition, rehabilitation is the primary treatment for ACL injury, but this study did not evaluate the compliance with rehabilitation programmes or the rehabilitation programmes that were performed, which might influence the results both negatively and positively. Moreover, associated meniscal or cartilage injuries are associated with a lower rate of RTS, which was not controlled for in this study and could affect the outcome [66]. Lastly, due to the many analysed variables, in combination with the significance level of 0.05, there is a risk of type-I errors. To account for this potential limitation, Cohen’s d was added. Cohen’s d is a measurement of effect size to further interpret results. As a result, due to the above-mentioned limitations, the results from the presented study should be interpreted with caution.

## Conclusion

A smaller proportion of patients with GJH return to knee-strenuous sports compared with patients without GJH during the first two years after ACL reconstruction. No differences in clinical outcomes were found between patients with and without GJH.

### Supplementary Information

**Additional file 1.**

## Data Availability

The dataset used and/or analyzed during the current study are available from the corresponding author in response to a reasonable request.

## Abbreviations

ACL: Anterior cruciate ligament
GJH: Generalized joint hypermobility
HT: Hamstring tendon
PT: Patella tendon
QT: Quadriceps tendon
PROs: Patient-reported outcomes
RTS: Return to sport
2KI: Second knee injury
ACL-RSI: ACL-Return to Sport after Injury scale
RTP: Return to pre-injury level of activity
ICC: Intraclass correlation coefficient
ME: Maximum effort
LSI: Limb symmetry index
Tegner: Tegner Activity Scale
K-SES: Knee Self-Efficacy Scale
KOOS: Knee injury and Osteoarthritis Outcome Score
QoL: Quality of life
HE: Hyperextension
n: Number
kg: Kilogram
cm: Centimeters
SD: Standard deviation
Min: Minimum
Max: Maximum
CI: Confidence interval
