# Supplementary material for: Lower rates of return to sport in patients with generalised joint hypermobility two years after ACL reconstruction: a prospective cohort study
Source: BMC Sports Sci Med Rehabil. 2023 Aug 12;15:100. doi: 10.1186/s13102-023-00707-2 (PMC10422717; doi:10.1186/s13102-023-00707-2)
Supplement: Supplementary file 1 — Additional file 1. [file 13102_2023_707_MOESM1_ESM.docx]

**Appendix**

| Table S1 – Number of patients who performed muscle function tests and patient-reported outcomes at RTS and RTP. | | | | | | |
| --- | --- | --- | --- | --- | --- | --- |
|  | **RTS** | | | **RTP** | | |
| Muscle function tests, n | **Total** | **GJH** | **Non-GJH** | **Total** | **GJH** | **Non-GJH** |
| Quadriceps strength | 590 | 80 | 510 | 443 | 65 | 378 |
| Hamstring strength | 590 | 80 | 510 | 443 | 65 | 378 |
| Vertical hop | 493 | 66 | 427 | 329 | 47 | 282 |
| Hop for distance | 497 | 66 | 431 | 339 | 48 | 291 |
| Side hop | 480 | 59 | 421 | 319 | 41 | 278 |
| Patient-reported outcomes, n | **Total** | **GJH** | **Non-GJH** | **Total** | **GJH** | **Non-GJH** |
| K-SES | 665 | 89 | 577 | 520 | 79 | 441 |
| KOOS_Pain_ | 662 | 89 | 573 | 519 | 79 | 440 |
| KOOS_Symptom_ | 662 | 89 | 573 | 519 | 79 | 440 |
| KOOS_Sports_ | 662 | 89 | 573 | 519 | 79 | 440 |
| KOOS_QoL_ | 662 | 89 | 573 | 519 | 79 | 440 |
| ACL-RSI | 542 | 73 | 469 | 399 | 57 | 342 |
| n = number of patients, RTS = Return to Sport, RTP = Return to Pre-injury level of activity, GJH = Generalised Joint Hypermobility, LSI = Limb Symmetry Index, SD = standard deviation, K-SES = Knee Self-Efficacy Scale, KOOS = Knee injury and Osteoarthritis Outcome Score, QoL = Quality of Life, ACL-RSI = ACL – Return to Sport after Injury Scale | | | | | | |

| Table S2 – Results in muscle function tests and patient-reported outcomes at the time of RTS and RTP. | | | | | | |
| --- | --- | --- | --- | --- | --- | --- |
|  | **RTS** | | | **RTP** | | |
| Muscle function tests  LSI %, mean ± SD (n missing) | **GJH** | **Non-GJH** | **p-value** | **GJH** | **Non-GJH** | **p-value** |
| Quadriceps strength | 91.1 ± 14.2 (10) | 92.7 ± 12.5 (72) | 0.286 | **87.3 ± 13.5 (15)**  **(95% CI 83.9 – 90.5)** | **91.7 ± 14.3 (68)**  **(95% CI 90.2 – 93.1)** | **0.022*** |
| Hamstring strength | 98.7 ± 11.2 (10) | 99.0 ± 13.3 (72) | 0.840 | 99.1 ± 11.9 (15) | 98.5 ± 12.5 (68) | 0.729 |
| Vertical hop | 91.5 ± 16.3 (24) | 90.4 ± 15.8 (155) | 0.596 | 88.9 ± 15.9 (33) | 88.3 ± 17.3 (164) | 0.566 |
| Hop for distance | 96.3 ± 10.7 (24) | 94.6 ± 9.7 (151) | 0.124 | 92.6 ± 10.8 (32) | 93.6 ± 11.2 (155) | 0.555 |
| Side hop | 99.1 ± 17.4 (31) | 96.0 ± 16.2 (161) | 0.174 | 100.8 ± 17.3 (39) | 96.1 ± 17.8 (168) | 0.117 |
| Patient-reported outcomes  Mean ± SD (n missing) | **GJH** | **Non-GJH** | **p-value** | **GJH** | **Non-GJH** | **p-value** |
| K-SES | 8.3 ± 1.5 (1) | 8.4 ± 1.6 (5) | 0.436 | 7.8 ± 1.9 (1) | 8.2 ± 1.8 (5) | 0.114 |
| KOOS_Pain_ | 88.8 ± 10.4 (1) | 88.2 ± 11.8 (9) | 0.667 | 87.6 ± 12.0 (1) | 87.2 ± 12.8 (6) | 0.794 |
| KOOS_Symptom_ | 82.1 ± 14.0 (1) | 79.9 ± 15.6 (9) | 0.208 | 79.9 ± 15.6 (1) | 78.6 ± 16.8 (6) | 0.516 |
| KOOS_Sports_ | 71.7 ± 21.4 (1) | 74.4 ± 20.1 (9) | 0.254 | 68.0 ± 25.6 (1) | 72.2 ± 22.6 (6) | 0.135 |
| KOOS_QoL_ | 58.3 ± 17.1 (1) | 62.3 ± 18.6 (9) | 0.057 | 58.8 ± 17.8 (1) | 62.4 ± 20.4 (6) | 0.145 |
| ACL-RSI | 62.0 ± 20.2 (1) | 66.5 ± 18.3 (27) | 0.053 | 68.2 ± 18.7 (1) | 69.7 ± 18.6 (14) | 0.573 |
| n = number of patients, RTS = Return to Sport, RTP = Return to Pre-injury level of activity, GJH = Generalised Joint Hypermobility, LSI = Limb Symmetry Index, SD = standard deviation, K-SES = Knee Self-Efficacy Scale, KOOS = Knee injury and Osteoarthritis Outcome Score, QoL = Quality of Life, ACL-RSI = ACL – Return to Sport after Injury Scale, CI = confidence interval, * = statistically significant difference (p<0.05) | | | | | | |
